# Limited Interchangeability of Smartwatches and Lace-Mounted IMUs for Running Gait Analysis

**DOI:** 10.3390/s25175553

**Published:** 2025-09-05

**Authors:** Theodor Meingast, Bryson Carrier, Amanda Melvin, Kenneth M. Kozloff, Alexandra F. DeJong Lempke, Adam S. Lepley

**Affiliations:** 1 School of Kinesiology, University of Michigan, Ann Arbor, MI 48109, USA; tmein@umich.edu (T.M.); brysonca@umich.edu (B.C.); acmelvin@umich.edu (A.M.); kenkoz@umich.edu (K.M.K.); 2 Department of Kinesiology and Nutrition Sciences, University of Nevada-Las Vegas, Las Vegas, NV 89154, USA; 3 School of Medicine, Department of Physical Medicine and Rehabilitation, Virginia Commonwealth University, Richmond, VA 23284, USA; alexandra.lempke@vcuhealth.org

**Keywords:** wearable sensors, biomechanics, gait, field-based assessment, biometric technology, fitness tracker, activity monitor

## Abstract

Spatiotemporal running metrics such as cadence, stride length (SL), and ground contact time (GCT) are important for assessing performance and injury risk. However, such metrics are traditionally assessed using laboratory-based tools that are often inaccessible in applied settings. Wearable devices including smartwatches and lace-mounted inertial measurement units (IMUs) offer promising alternatives, yet cross-device agreement in reporting spatiotemporal variables remains unclear. This study evaluated agreement between a commercial smartwatch and lace-mounted IMUs across varied distances and environments in 65 physically active adults (33 female/32 male, height: 171.0 ± 8.9 cm; weight: 70.9 ± 15.2 kg). Participants completed indoor and outdoor runs (2.5 km, 5 km, 10 km, 20 km) wearing both devices simultaneously. Average cadence demonstrated acceptable agreement (MAPE = 4.1%, CCC = 0.66) and supported equivalence, particularly among males, during outdoor conditions, and longer run distances. In contrast, peak cadence showed weak correlation (MAPE = 5.3%, CCC = 0.29), and SL and GCT demonstrated poor agreement (MAPE = 14.9–19.0%, CCC = 0.30–0.39) across all conditions. While average cadence may serve as a metric for cross-device comparisons, especially for males, and longer-distance outdoor runs, other spatiotemporal metrics demonstrated poor agreement, limiting interchangeability. Understanding device-specific capabilities is essential when interpreting wearable-derived gait data. Further validation using gold-standard tools is needed to support accurate and applied use of wearable technologies.

## 1. Introduction

Spatiotemporal running biomechanics, including ground contact time (GCT), cadence, and stride length (SL), play a crucial role in assessing running performance and injury risk. These metrics have been shown to serve as key indicators of fatigue, and are often used as indicators of biomechanical efficiency, particularly during prolonged endurance events [1,2,3]. For example, shorter GCT, increased cadence, and optimized SL have been associated with improved endurance performance [4,5]. Additionally, alterations in spatiotemporal variables have been linked to injury susceptibility. Increased GCT, longer SL, and decreased cadence have been associated with the development of exercise-related lower leg pain, while decreased cadence and increased SL have been identified as risk factors for bone stress injuries [6,7,8,9,10]. Given the key role of spatiotemporal parameters in running biomechanical profiles, ensuring accessible and accurate measurement of these variables is crucial for individuals seeking to assess running performance and running-related injury.

The criterion standard for measuring spatiotemporal variables requires 3-dimensional (3-D) motion capture systems, force plates, and high-speed video analysis in controlled laboratory environments. While these methods provide good accuracy and reliability, these systems are cost-prohibitive, time-intensive, and largely inaccessible to recreational runners and clinicians who wish to monitor performance and injury risk in outdoor settings. This limitation has driven interest in wearable technology as an alternative for field-based running assessments [11]. Advancements in wearable sensor technologies, such as improvements in sampling rates, integration of inertial measurement units (IMU) with global positioning systems (GPS) and other sensors, and unique device placements, have enabled runners to monitor biomechanical data in real time. Among commercially available devices, lace-mounted IMUs can provide detailed kinetic, kinematic, and spatiotemporal variables during running. Previous studies [12,13] have validated the accuracy of the lace-mounted RunScribe^TM^ IMU sensors against laboratory-based standards, reporting minimal mean differences and excellent agreement for spatiotemporal variables (cadence: ICC = 0.97, r = 0.93; SL: ICC = 0.80–0.86, MAE = 0.7–0.8 m; GCT: ICC = 0.92–0.93, MAE = 27–29 ms) [12,14,15]. Additionally, these devices have demonstrated sensitivity in determining changes in spatiotemporal biomechanics in response to varying speeds, surfaces, and intentional spatiotemporal gait-training modifications [2,16]. These findings suggest that lace-mounted IMUs serve as an accurate and practical tool for consumer use in monitoring running biomechanics.

Conversely, smartwatches offer a more convenient, affordable, and widely adopted wearable device that could serve as an alternative for tracking running biomechanics. However, smartwatch validation in accurately measuring spatiotemporal variables is limited and has yielded mixed findings. Some studies suggest smartwatches can detect variability in gait patterns but have demonstrated mixed error and agreement statistics when estimating SL during gait using Garmin, Apple and Samsung smartwatch devices (RSME = 5.29 cm; MAE = 0.13 m; ICC = 0.60) [17,18,19]. Additionally, the ability to monitor dynamic changes in running biomechanics remains uncertain. For example, an investigation using a Garmin smartwatch was not able to detect alterations in spatiotemporal variables when participants intentionally modified their running styles, raising questions about measurement sensitivity [20].

Despite the increasing availability of wearable devices for runners, no study, to our knowledge, has directly assessed the agreement between lace-mounted IMUs and smartwatches in reporting spatiotemporal variables during running activities. The level of agreement between consumer wearable devices has direct implications for applied use. Establishing whether two commonly used consumer technologies, positioned at different anatomical sites, provide comparable data is essential, as discrepancies could influence how athletes, coaches, and clinicians interpret performance or injury risk. A clear understanding of device agreement helps ensure that training and rehabilitation decisions are based on accurate, reliable information. Therefore, the purpose of this study was to evaluate the agreement between lace-mounted IMUs and a commercially available smartwatch for measuring spatiotemporal variables during running across different distances and environments. Establishing the level of agreement between these devices could improve our understanding of the interchangeability, accuracy, and real-world application of wearable devices in running assessments.

## 2. Materials and Methods

The data presented in this study is a subset of an overall dataset focused on examining wearable technology in physically active populations. Adult participants over the age of 18 were recruited from a university population and surrounding community and were included if they self-reported participating in moderate to vigorous physical activity at least three days per week. Participants were excluded if they had a contraindication to intense exercise (e.g., cardiovascular disease, significant musculoskeletal or neurological impairments, etc.) or were pregnant at the time of testing. All participants provided written informed consent prior to testing, and all procedures were approved by the University’s Institutional Review Board (IRB#: HUM00220366).

All participant demographic information was collected at baseline through electronic surveys (REDCap, Vanderbilt University, Nashville, TN, USA). Participants then completed three testing sessions, each separated by one to two weeks (days between visit one and two: 11.0 ± 5.4; between visit two and three: 11.4 ± 6.7). Each participant completed an outdoor 5 km run at visit one, an indoor 5 km run at visit two, and was assigned to either an indoor or outdoor run of variable distance (2.5 km, 10 km, 20 km) for their third visit. Cohort determinations for visit three were based on self-reported running experience (days per week engaged in running activity) and self-reported ability to complete the distance at the time of initial study participation screening. All participants were instructed to complete each run at a self-selected light to somewhat hard pace (RPE 11–13) using the Borg Rating of Perceived Exertion scale [21].

**Figure 1:**
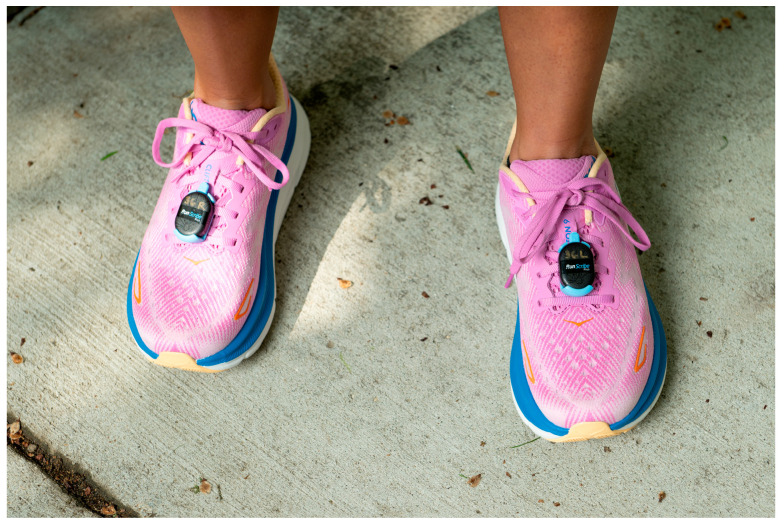
Image showing placement of bilateral lace-mounted IMUs, secured with the specific lace cradles at the midfoot of the running shoes.

During all trials, participants wore a smartwatch (Apple Watch series 7, watchOS 9, Apple Inc., Cupertino, CA, USA, sampling frequency: not reported) on their left wrist, and bilateral lace-mounted IMUs (RunScribe Pods, Scribe Labs, Moss Beach, CA, USA, sampling frequency: 250 Hz) secured using the sensor-specific lace cradles at the midfoot on their running shoes (Figure 1). This study was designed as a direct comparison between the two devices to evaluate their agreement in measuring spatiotemporal running variables. Importantly, we did not aim to validate these devices against a criterion measure (e.g., 3D motion capture or force plates); therefore, the results should be interpreted solely as a comparative analysis of cross-device agreement. Investigators ensured proper fit of each device per manufacturer instructions so that the devices did not move excessively during activity. Investigators reset device settings and created individual profiles using the profile settings for each device prior to each assessment, including participant date of birth, sex, height, and weight. The lace-mounted IMUs were additionally calibrated to positioning on the foot immediately prior to each running trial. All indoor trials were completed on a motorized treadmill (4Front, Woodway, Waukesha, WI, USA). All outdoor runs were completed on pre-determined GPS-measured routes with standardized start and stop locations and consisted of loops around a university campus that aimed for continuous running and limited road intersections. All outdoor runs were completed on concrete sidewalks, with elevation gains of approximately 7 m for the 2.5 km and 5 km courses and approximately 105 m for the 10 km and 20 km courses. Prior to the outdoor runs, participants were made familiar with the route and were remotely monitored by lab staff during their run via a tracking device (AirTag, Apple Inc., Cupertino, CA, USA).

Spatiotemporal variables across all runs, including average GCT (ms), peak and average cadence (steps/minute), and average SL (m), were exported from each device’s exercise recording app following each trial. Per developer documentation [22,23,24], SL is reported differently between devices, as the smartwatch reports stride length as step length, or half of the defined SL. Thus, smartwatch SL values were corrected (eq. smartwatch SL = reported step length × 2) to account for this difference.

### Data Analysis

For all trials, overall agreement was evaluated via tests of error, linearity, and equivalence between the lace-mounted IMUs and smartwatch for each variable. Mean absolute error (MAE) and mean absolute percentage error (MAPE) were calculated for error analysis. Linearity was established via Lin’s Concordance Correlation Coefficient (CCC), Pearson’s Product Moment Correlation (r), and Deming Regression. Correlation coefficients were interpreted as follows: 0 to <0.2, very weak; ≥0.2 to <0.4, weak; ≥0.4 to <0.6, moderate; ≥0.6 to <0.8, strong; and ≥0.8 to 1.0, very strong [25]. Equivalence testing was performed via confidence interval for difference in means, using the 90% confidence interval from paired t-tests and 10% (±5%) of the criterion mean as the equivalence window [26]. A 10% equivalence window was selected to align with prior wearable validation research where it is widely applied for cross-study comparisons, and because 5–10% changes in spatiotemporal variables, specifically step rate, are known to produce clinically meaningful alterations in lower-extremity loading and running kinematics [27,28,29,30]. Binary results for equivalence testing are presented as “Supported” or “Not Supported.” In addition, combined agreement criteria were set at MAPE < 10%, CCC > 0.7, and equivalence supported at 10% (±5%) of the criterion mean for the equivalence window, based on the 90% CI [31].

Data were further stratified by sex, distance, and environment for additional analyses (Table 1, Table 2 and Table 3). Environment was categorized by indoor and outdoor trials and distance presented as short (2.5 km, 5 km) and long-distance trials (10 km, 20 km). Supplemental stratifications were also conducted on height (<166 cm, 166–175 cm, >175 cm) and weight (<60 kg, 60–70 kg, 70–80 kg, 80–90 kg, >90 kg)

## 3. Results

A total of 65 participants (33 female/32 male, height: 171.0 ± 8.9 cm; body mass: 70.9 ± 15.2 kg) were included. There was a total of 192 running trials that were captured by the smartwatch and 191 by the lace-mounted IMU sensors and included in this study. Discrepancies in sample size across analyses reflect instances where one device did not report a given variable, and these trials were therefore excluded from that pairwise comparison. The number of runs included for each analysis can be found in Table 1, Table 2 and Table 3.

Overall device agreement for average cadence between the smartwatch and lace-mounted IMU sensors demonstrated less than 10% error between devices (MAPE: 4.1%). Correlation coefficients were moderate (r = 0.74; CCC = 0.66), and equivalency testing was supported (Table 1). Stratifications for average cadence revealed that agreement between devices was greater for males, longer distances, and outdoor trials, as these analyses met accuracy thresholds of MAPE < 10%, CCC > 0.7, and equivalence supported at 10% (±5%) of the criterion mean (Table 1, Table 2 and Table 3).

There was mixed agreement between devices for peak cadence. Although measurement error remained low for overall data (MAPE < 10% across all stratifications), correlation values were considered weak (r = 0.45; CCC = 0.29), and equivalence was not supported (Table 1). Sex, distance, and environment stratifications did not influence accuracy statistics (Table 1, Table 2 and Table 3).

**Figure A1:**
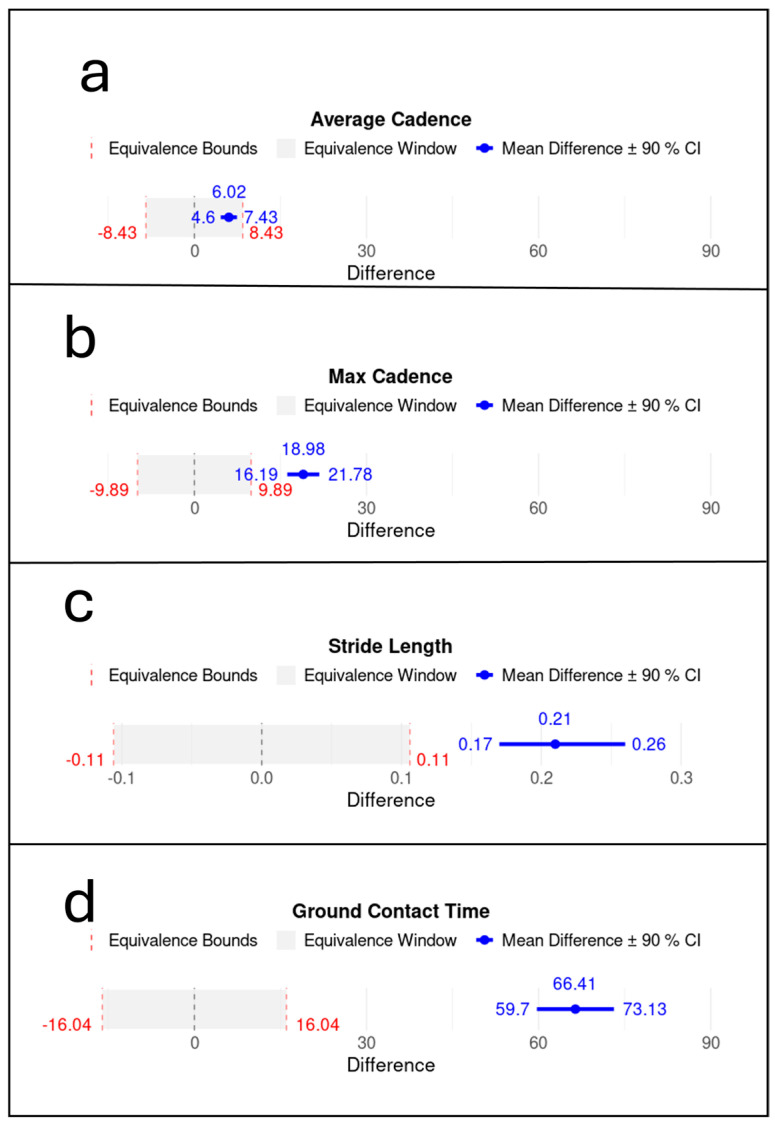
Equivalency plots for the overall cohort for average cadence (**a**), peak cadence (**b**), stride length (**c**), and ground contact time (**d**) for lace-mounted IMUs compared to smartwatch measurement. Equivalence window for equivalence plots determined based on 10% of criterion mean (±5%).

**Figure A2:**
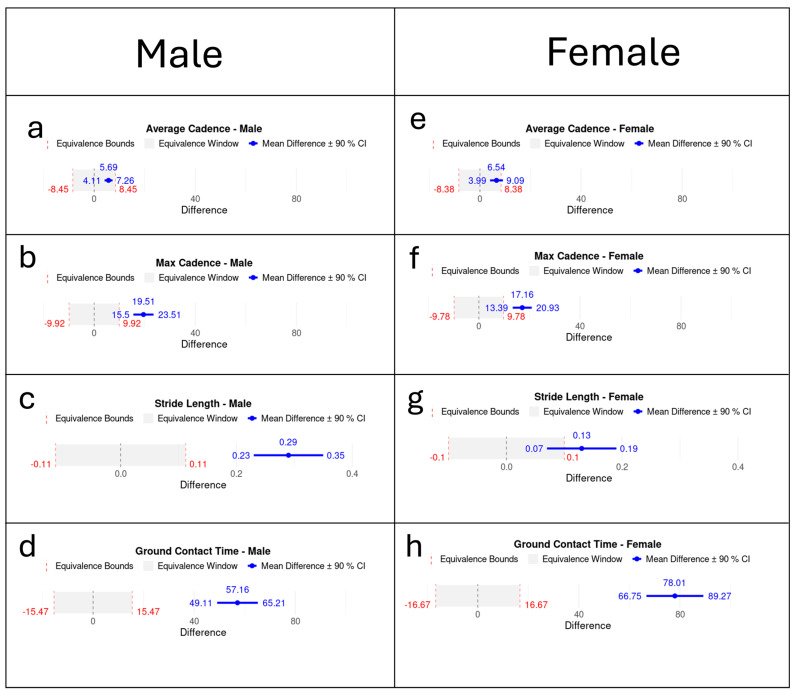
Equivalency plots, stratified by males (**a**–**d**) and females (**e**–**h**) for average cadence (**a**,**e**), peak cadence (**b**,**f**), stride length (**c**,**g**), and ground contact time (**d**,**h**) for lace-mounted IMUs compared to smartwatch measurement. Equivalence window for equivalence plots determined based on 10% of criterion mean (±5%).

**Figure A3:**
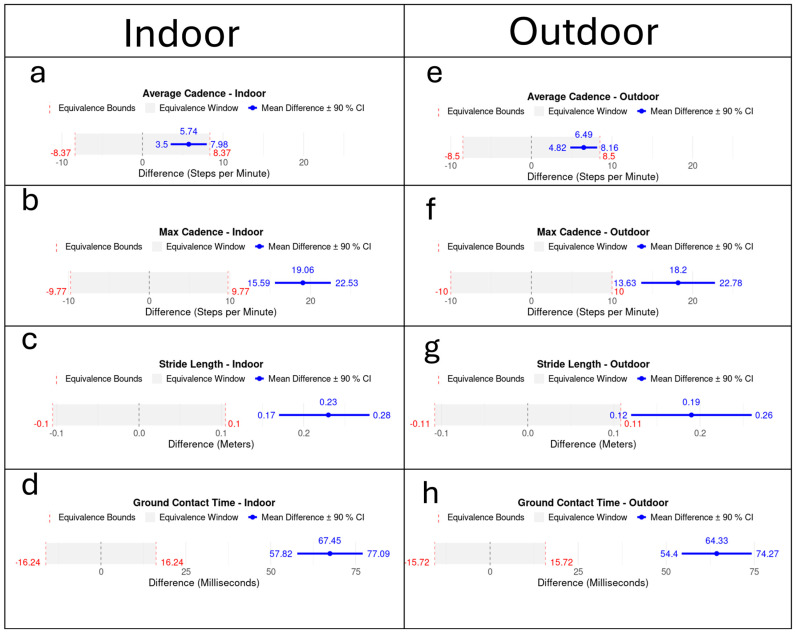
Equivalency plots, stratified by indoor (**a**–**d**) and outdoor (**e**–**h**) trials for average cadence (**a**,**e**), peak cadence (**b**,**f**), stride length (**c**,**g**), and ground contact time (**d**,**h**) for lace-mounted IMUs compared to smartwatch measurement. Equivalence window for equivalence plots determined based on 10% of criterion mean (±5%).

**Figure A4:**
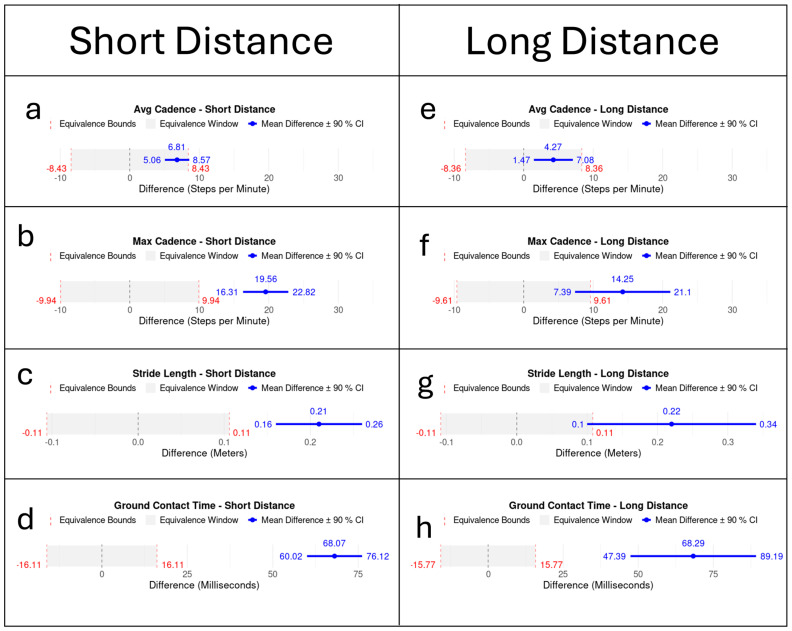
Equivalency plots, stratified by short (2.5 km and 5 km); (**a**–**d**) and long (10 km and 20) km; (**e**–**h**) distance trials for average cadence (**a**,**e**), peak cadence (**b**,**f**), stride length (**c**,**g**), and ground contact time (**d**,**h**) for lace-mounted IMUs compared to smartwatch measurement. Equivalence window for equivalence plots determined based on 10% of criterion mean (±5%).

**Figure A5:**
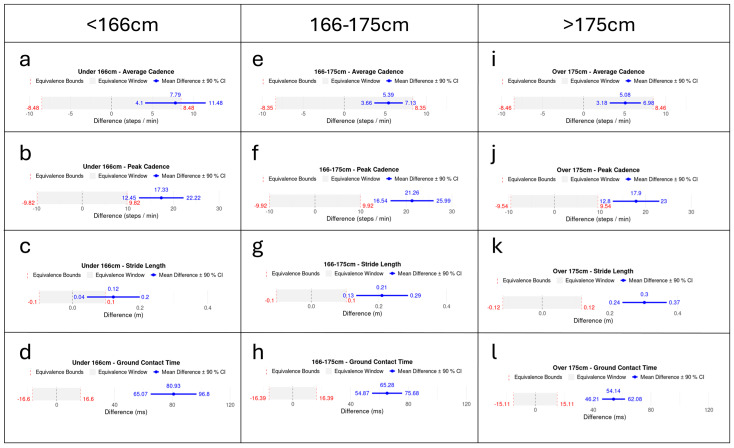
Equivalency plots, stratified by height (<166 cm (**a**–**d**); 166–175 cm (**e**–**h**); >175 cm (**i**–**l**)) for average cadence (**a**,**e**,**i**), peak cadence (**b**,**f**,**j**), stride length (**c**,**g**,**k**), and ground contact time (**d**,**h**,**l**) for lace-mounted IMUs compared to smartwatch measurement. Equivalence window for equivalence plots determined based on 10% of criterion mean (±5%).

**Figure A6:**
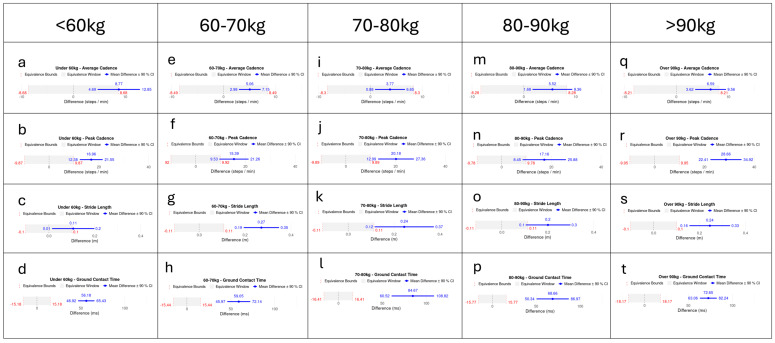
Equivalency plots, stratified by weight (<60 kg (**a**–**d**); 60–70 kg (**e**–**h**); 70–80 kg (**i**–**l**); 80–90 kg (**m**–**p**); >90 kg (**q**–**t**) for average cadence (**a**,**e**,**i**,**m**,**q**), peak cadence (**b**,**f**,**j**,**n**,**r**), stride length (**c**,**g**,**k**,**o**,**s**), and ground contact time (**d**,**h**,**l**,**p**,**t**) for lace-mounted IMUs compared to smartwatch measurement. Equivalence window for equivalence plots determined based on 10% of criterion mean (±5%).

There was poor agreement overall for SL (corrected SL for smartwatch) and GCT metrics, with poor error rates (MAPE range = 14.9–19.0%), weak-to-moderate correlation coefficients (r range = 0.51–0.61; CCC = 0.30–0.39), and unsupported equivalence testing (Table 1). Sex, distance, and environment did not influence interpretation of agreement statistics for SL and GCT (Table 1, Table 2 and Table 3). Equivalence plots and supplemental stratifications for height and weight can be found in Appendix A in Figure A1, Figure A2, Figure A3, Figure A4, Figure A5 and Figure A6.

**Table 1:** Agreement statistics between lace-mounted IMUs and smartwatch for spatiotemporal variables for all data and gender stratified comparisons.

		n	Device	Average (SD)	MAE	MAPE	r	CCC	Deming Intercept/Slope
Overall	* AC (step /min)	191	Lace-mounted IMU	169.1 (13.3)	—	—	—	—	—
		192	Smartwatch	162.8 (16.2)	6.8	4.1%	0.74	0.66	−72.4/1.39
	PC (step /min)	191	Lace-mounted IMU	198.7 (25.0)	—	—	—	—	—
		190	Smartwatch	178.7 (15.9)	20.5	9.5%	0.45	0.29	95.2/0.42
	SL (m)	190	Lace-mounted IMU	2.12 (0.39)	—	—	—	—	—
		190	Smartwatch	1.90 (0.28)	0.32	14.4%	0.51	0.39	0.86/0.49
	GCT (ms)	191	Lace-mounted IMU	318.1 (69.9)	—	—	—	—	—
		190	Smartwatch	254.5 (32.1)	66.6	19.0%	0.67	0.30	143.59/0.35
Female	AC (step /min)	86	Lace-mounted IMU	168.3 (13.9)	—	—	—	—	—
		88	Smartwatch	161.4 (17.6)	7.7	4.6%	0.65	0.58	−92.7/1.51
	PC (step /min)	86	Lace-mounted IMU	197.3 (23.5)	—	—	—	—	—
		87	Smartwatch	178.4 (12.2)	18.3	8.6%	0.43	0.25	116.8/0.32
	SL (m)	86	Lace-mounted IMU	1.99 (0.32)	—	—	—	—	—
		87	Smartwatch	1.86 (0.28)	0.28	13.4%	0.34	0.31	0.57/0.65
	GCT (ms)	86	Lace-mounted IMU	330.2 (74.3)	—	—	—	—	—
		87	Smartwatch	255.8 (30.6)	78.3	21.4%	0.61	0.23	159.3/0.29
	* AC (step /min)	102	Lace-mounted IMU	169.5 (12.8)	—	—	—	—	—
Male		101	Smartwatch	163.6 (15.4)	6.1	3.7%	0.82	0.74	-57.0/1.3
	PC (step /min)	102	Lace-mounted IMU	198.9 (25.4)	—	—	—	—	—
		100	Smartwatch	178.8 (18.7)	21.4	9.9%	0.47	0.33	70.0/0.55
	SL (m)	101	Lace-mounted IMU	2.25 (0.40)	—	—	—	—	—
		100	Smartwatch	1.95 (0.27)	0.36	15.5%	0.57	0.37	0.92/0.46
	GCT (ms)	102	Lace-mounted IMU	307.4 (65.5)	—	—	—	—	—
		100	Smartwatch	252.5 (33.7)	57.4	17.1%	0.74	0.37	125.0/0.41

Abbreviations: AC, average cadence; PC, peak cadence; SL, stride length; GCT, ground contact time; MAE, mean absolute error; MAPE, mean absolute percent error; r, Pearson Correlation Coefficient; CCC, Lin’s Concordance Correlation Coefficient. * Indicates that equivalency testing was supported between devices.

**Table 2:** Agreement statistics between lace-mounted IMUs and smartwatch for spatiotemporal variables stratified by distance.

		n	Device	Average (SD)	MAE	MAPE	r	CCC	Deming Intercept/Slope
Short Distance	AC (step /min)	143	Lace-mounted IMU	169.1 (13.7)	—	—	—	—	—
		143	Smartwatch	162.2 (17.3)	7.6	4.5%	0.71	0.63	−74.17/1.4
	PC (step /min)	143	Lace-mounted IMU	199.6 (25.1)	—	—	—	—	—
		141	Smartwatch	179.2 (16.6)	21.5	9.9%	0.46	0.30	88.32/0.46
	SL (m)	142	Lace-mounted IMU	2.13 (0.39)	—	—	—	—	—
		141	Smartwatch	1.90 (0.28)	0.3	14.8%	0.46	0.36	0.9/0.47
	GCT (ms)	143	Lace-mounted IMU	319.6 (73.0)	—	—	—	—	—
		141	Smartwatch	254.5 (33.1)	68.3	19.3%	0.69	0.3	142.09/0.35
Long Distance	* AC (step /min)	25	Lace-mounted IMU	167.7 (12.1)	—	—	—	—	—
		26	Smartwatch	163.1 (14.8)	4.2	2.5%	0.83	0.78	−44.9/1.24
	PC (step /min)	25	Lace-mounted IMU	194.5 (22.9)	—	—	—	—	—
		26	Smartwatch	178.2 (14.7)	15.2	7.2%	0.41	0.29	77.12/0.52
	SL (m)	25	Lace-mounted IMU	2.14 (0.41)	—	—	—	—	—
		26	Smartwatch	1.92 (0.30)	0.3	14.7%	0.54	0.37	1.15/0.37
	GCT (ms)	25	Lace-mounted IMU	315.8 (68.0)	—	—	—	—	—
		26	Smartwatch	246.5 (26.0)	68.5	19.4%	0.55	0.18	182.82/0.2

Abbreviations: AC, average cadence; PC, peak cadence; SL, stride length; GCT, ground contact time; MAE, mean absolute error; MAPE, mean absolute percent error; r, Pearson Correlation Coefficient; CCC, Lin’s Concordance Correlation Coefficient. Short distance includes all 2.5 km and 5 km run trails (both indoor and outdoor). Long distance includes all 10 km and 20 km run trails (both indoor and outdoor). * Indicates that equivalency testing was supported between devices.

**Table 3:** Agreement statistics between lace-mounted IMUs and smartwatch for spatiotemporal variables stratified by indoor or outdoor environment.

		n	Device	Average (SD)	MAE	MAPE	r	CCC	Deming Intercept/Slope
Indoor	AC (step /min)	100	Lace-mounted IMU	168.2 (13.0)	—	—	—	—	—
		99	Smartwatch	162.0 (17.6)	6.5	3.9%	0.67	0.6	−105.97/1.6
	PC (step /min)	100	Lace-mounted IMU	195.9 (22.2)	—	—	—	—	—
		97	Smartwatch	176.6 (12.1)	19.2	9.1%	0.44	0.24	115.47/0.31
	SL (m)	99	Lace-mounted IMU	2.11 (0.35)	—	—	—	—	—
		97	Smartwatch	1.86 (0.27)	0.3	13.1%	0.5	0.36	0.83/0.5
	GCT (ms)	100	Lace-mounted IMU	321.2 (69.6)	—	—	—	—	—
		97	Smartwatch	257.0 (33.0)	67.8	19.0%	0.63	0.28	148.4/0.34
Outdoor	* AC (step /min)	86	Lace-mounted IMU	170.3 (13.6)	—	—	—	—	—
		87	Smartwatch	163.6 (15.2)	7.0	4.1%	0.82	0.74	−35.06/1.17
	PC (step /min)	86	Lace-mounted IMU	201.5 (27.0)	—	—	—	—	—
		87	Smartwatch	181.2 (19.2)	21.4	9.8%	0.44	0.32	75.03/0.53
	SL (m)	86	Lace-mounted IMU	2.15 (0.43)	—	—	—	—	—
		87	Smartwatch	1.97 (0.27)	0.3	15.8%	0.47	0.37	1.05/0.42
	GCT (ms)	86	Lace-mounted IMU	312.7 (69.9)	—	—	—	—	—
		87	Smartwatch	251.1 (29.8)	64.4	18.7%	0.70	0.30	147.24/0.33

Abbreviations: AC, average cadence; PC, peak cadence; SL, stride length; GCT, ground contact time; MAE, mean absolute error; MAPE, mean absolute percent error; r, Pearson Correlation Coefficient; CCC, Lin’s Concordance Correlation Coefficient. Indoor includes all run trials completed on indoor treadmill for all distances (2.5 km, 5 km, 10 km, 20 km). Outdoor includes all runs trials completed on outdoor routes for all distances (2.5 km, 5 km, 10 km, 20 km). * Indicates that equivalency testing was supported between devices.

## 4. Discussion

Wearable devices are increasingly used to monitor running biomechanics. However, the extent to which different devices, including those worn at the wrist vs. the foot, provide comparable spatiotemporal measurements during running remains uncertain. Establishing the level of agreement between these devices is essential for determining their interchangeability and ensuring accurate application of wearable-derived gait metrics in both research and applied settings. Thus, the current study evaluated the agreement between a smartwatch and lace-mounted IMU sensors for measuring key spatiotemporal running metrics, including cadence (average and peak), SL (corrected SL for smartwatch) and GCT, under varied environmental and distance conditions. Our findings revealed acceptable agreement in reported average cadence values between smartwatch and lace-mounted IMU sensors, particularly for males, longer distance runs, and outdoor trials. However, agreement between devices was poor for peak cadence, SL, and GCT regardless of sex, distance, or environment. These findings suggest that while smartwatches may offer sufficient agreement for monitoring average cadence in select contexts, there remains disagreement between devices in capturing more granular spatiotemporal variables. In applied settings where different devices, and specifically different anatomical locations, are used, caution is warranted when interpreting metrics beyond average cadence, especially in clinical or performance decision-making contexts.

Among all measured spatiotemporal variables, average cadence exhibited the strongest agreement between devices. Across overall, and sex-, distance-, and environment-stratified comparisons, average cadence consistently demonstrated acceptable MAPE and strong correlations, suggesting that both devices provided similar average cadence values at the provided thresholds. Equivalence testing further supported agreement for males, longer-distance runs, and outdoor trials; however, equivalence testing was not supported for females, shorter runs, or indoor trials. While the underlying reasons for these differences remain unclear, due in part to the proprietary nature of device algorithms, possible explanations include that algorithm development may have been based primarily on data from male participants in these conditions, or that device-specific methods account for confounding factors (such as sex, distance, or environment) in inconsistent ways. For example, greater agreement during outdoor compared to indoor trials may reflect the integration of GPS data in calculation of spatiotemporal variables [32,33]. Similarly, longer distance trials provide more overall data points, reducing variability and enhancing agreement for average cadence values between devices. For sex, differences in average cadence agreement may also highlight underlying biomechanical differences between males and females, potentially influencing how wrist-based sensors detect arm swing and estimate step frequency [34,35]. Particularly, females often display greater upper extremity motion, including greater trunk rotation and arm swing, which may influence wrist accelerometer signals and contribute to discrepancies between wrist- and shoe-mounted cadence estimates [36]. Although our methodology for outdoor trials does not allow us to directly compare device validation to a criterion standard, prior research has shown that both lace-mounted [12,13,37] and smartwatch [19,38] derived average cadence values demonstrate acceptable agreement with 3-D motion capture systems. Collectively, these findings support the utility of average cadence as a metric for cross-device comparisons in both controlled and applied settings.

Although average cadence demonstrated acceptable agreement, results were mixed for peak cadence. While there were relatively acceptable error rates between devices for peak cadence values, correlation coefficients remained weak, and equivalence testing was not supported in any condition. Averaging steps per minute across the entire trial likely reduces variability between devices and improves agreement for average cadence values, as this would minimize the influence of sensor noise/motion artifacts, momentary data loss, or sampling inconsistencies [39]. In contrast, peak cadence is a discrete value within the trial and thus may have been more susceptible to discrepancies arising from differences in sampling frequencies, filtering methods, and proprietary algorithmic definitions of what is considered the peak value, all of which are not publicly disclosed. There is also an inherent difference in cadence estimation via wrist-based sensors which estimates step frequency based on arm movement, as opposed to a two-pod sensor system that directly measures step-by-step movements at the feet, as biomechanical estimations have been found to vary with IMU sensor placements [40]. This discrepancy is likely to lead to differences in cadence and other spatiotemporal measurements between devices, and ultimately poorer sensitivity in estimations of step frequency, especially for discrete values such as peak cadence. These findings underscore the limitations in using wearable technologies interchangeably for measuring spatiotemporal outcomes.

Both SL (corrected SL for smartwatch) and GCT demonstrated poor agreement between devices, with poor error rates, weak-to-moderate correlations, and failure to pass equivalency testing. As opposed to cadence, which primarily requires consistent step detection across the trial duration by the device, SL and GCT are more complex spatiotemporal variables that rely on the accurate identification of discrete gait events (e.g., foot strike, toe-off) and the estimation of spatial displacement [41]. The inherent difference in sensor placement between devices (i.e., wrist vs. foot) likely contributes to significant differences in how each device derives SL and GCT variables. Although the smartwatch can use accelerometer data for arm movement and other movement events, different running forms and changes in arm swing during gait may make this difficult to apply broadly to a running population. Additionally, minor inaccuracies in distance or cadence calculations could significantly affect SL and GCT estimates, and these errors likely compound to produce poor overall estimations. By nature of placement, lace-mounted IMUs allow for vertical oscillation to be detected for each limb, eliminating the need to derive variables from arm motion. Previous literature suggests that lace-mounted IMUs are a valid means of assessing SL (ICC: 0.80–0.86; MAE: 0.7–0.8 m) and GCT (ICC: 0.92–0.93; MAE: 27–29 ms) [12,38], which have performed better compared to smartwatch-derived values [19]. However, without direct comparison to criterion 3-D motion capture testing in the current study, we are only able to conclude that there is no statistical agreement between devices and are unable to discern the validity of each device. The findings of the current study underscore the challenges in comparing higher-resolution gait metrics across different wearable platforms and highlight the need for careful consideration of device capabilities when selecting spatiotemporal measures in both research and applied settings [42,43].

Running intensity in the current study was standardized to self-selected pace using the Borg RPE scale to increase generalizability. However, this resulted in an inability to control for running speed across participants. Spatiotemporal variables are known to vary with running speed [44], and wearable devices may rely on speed-derived algorithms to estimate gait variables. Thus, variability in participant pace may have the potential to influence the agreement estimates between devices. However, both devices were worn simultaneously during each trial to experimentally minimize confounding effects of speed differences between participants. In addition, a post hoc paired t-test revealed no significant difference in running speed between the indoor (10.4 ± 2.1 km/h) and outdoor (10.6 ± 2.2 km/h) 5 km trials completed by all participants (t = 1.3, *p* = 0.19). Additionally, we did not use a criterion comparison (i.e., 3-D motion capture) for analysis. As a result, we were limited to assessing the agreement between the two devices, without the ability to determine criterion validity or which device, if either, introduced greater error. Future research should include a criterion reference device and examine device performance across varied distances, running speeds, and environments. Lastly, as mentioned previously, consumer wearable devices do not disclose proprietary algorithms used to determine the spatiotemporal variables reported in this study. Thus, an additional limitation is that we are unable to discern whether disagreement between devices arises from hardware/sensor or software/algorithm differences between devices. Similarly, an inherent limitation in working with wearable technology is that new models and software updates are regularly released. Our investigation kept the devices and software version constant to address this concern; however, regular testing and independent evaluation are important for the ever-evolving hardware and sensor changes, and software and algorithm updates in these devices.

## 5. Conclusions

The current study evaluated the agreement between a commercial smartwatch and lace-mounted IMUs for measuring spatiotemporal variables during running. While average cadence demonstrated acceptable agreement between devices and may serve as a metric for cross-device comparisons, especially for males, longer-distance runs, and outdoor trials, peak cadence, SL, and GCT demonstrated poor agreement, limiting their interchangeability. These findings highlight the importance of understanding device-specific capabilities and constraints when interpreting wearable-derived gait metrics. Continued validation work using criterion reference tools and across broader running conditions is essential to inform the appropriate use of wearable technology in both research and applied settings.

## Data Availability

The original contributions presented in this study are included in the article. Further inquiries can be directed to the corresponding author.

## Abbreviations

The following abbreviations are used in this manuscript:

SL	stride length
GCT	ground contact time
IMU	inertial measurement unit
MAPE	mean absolute percent error
GPS	global positioning system
MAE	mean absolute error
CCC	Lin’s Concordance Correlation Coefficient
